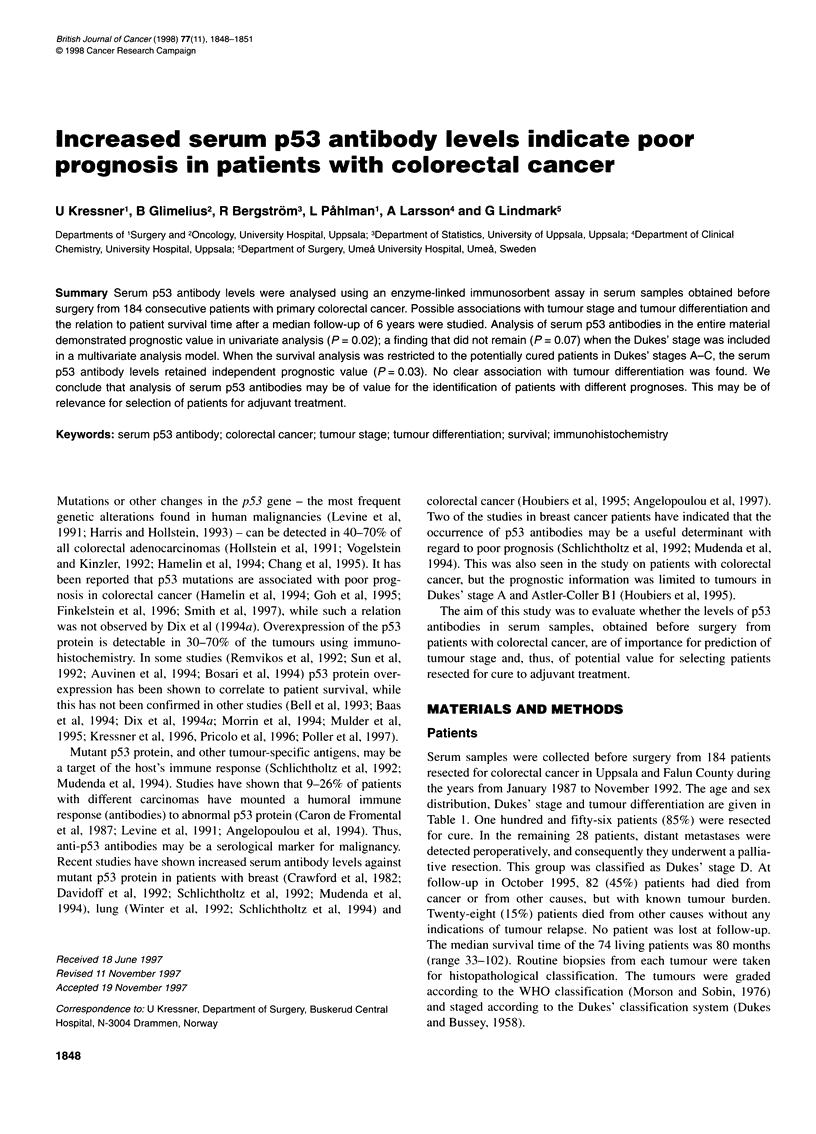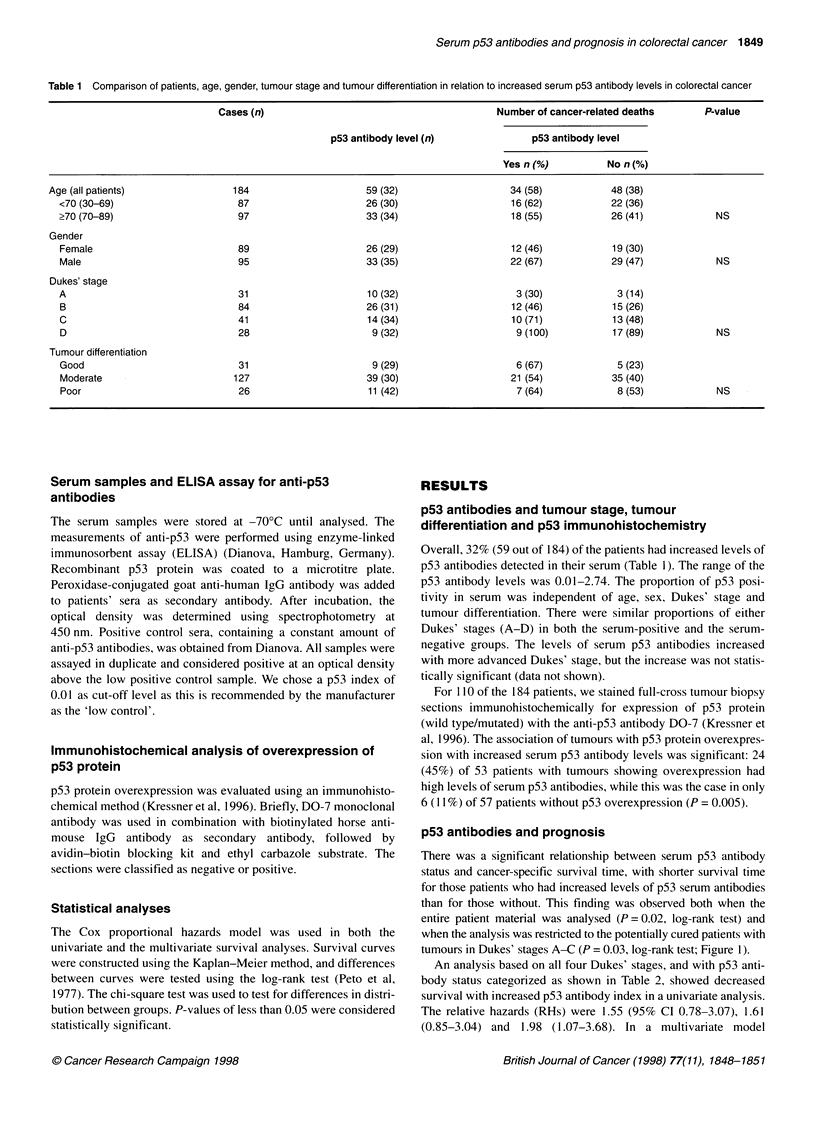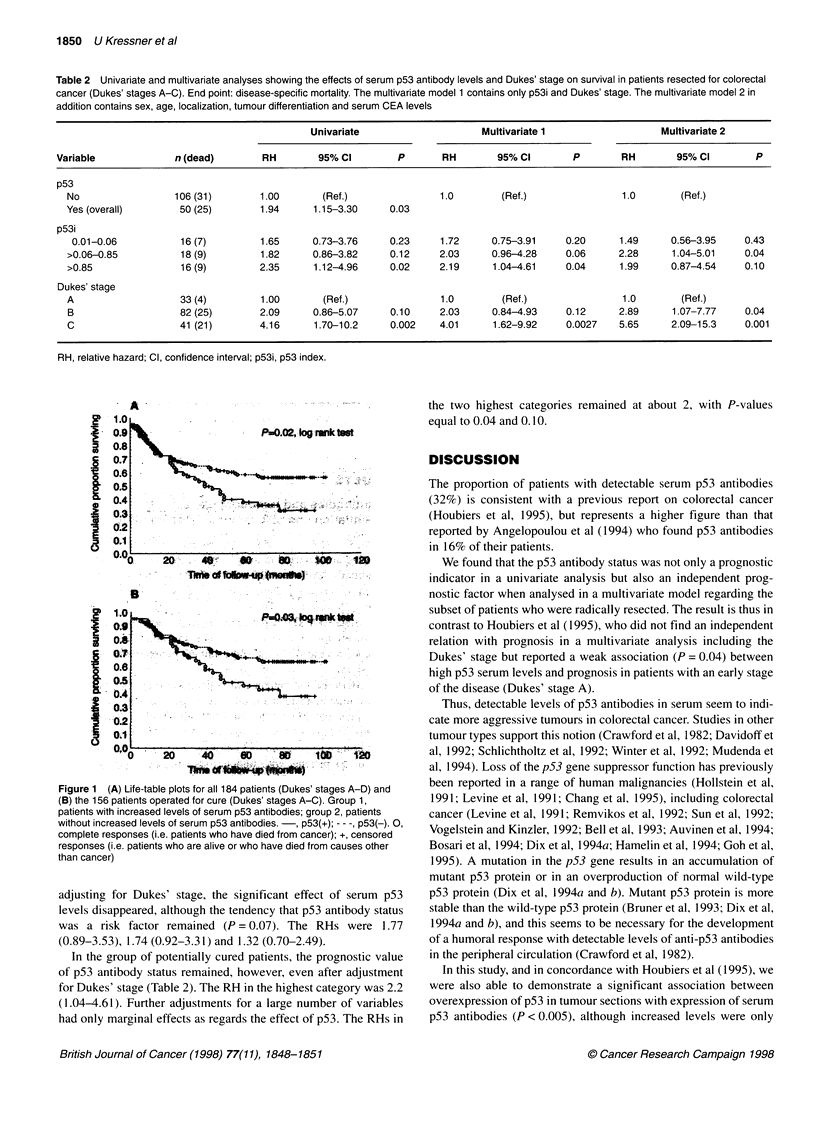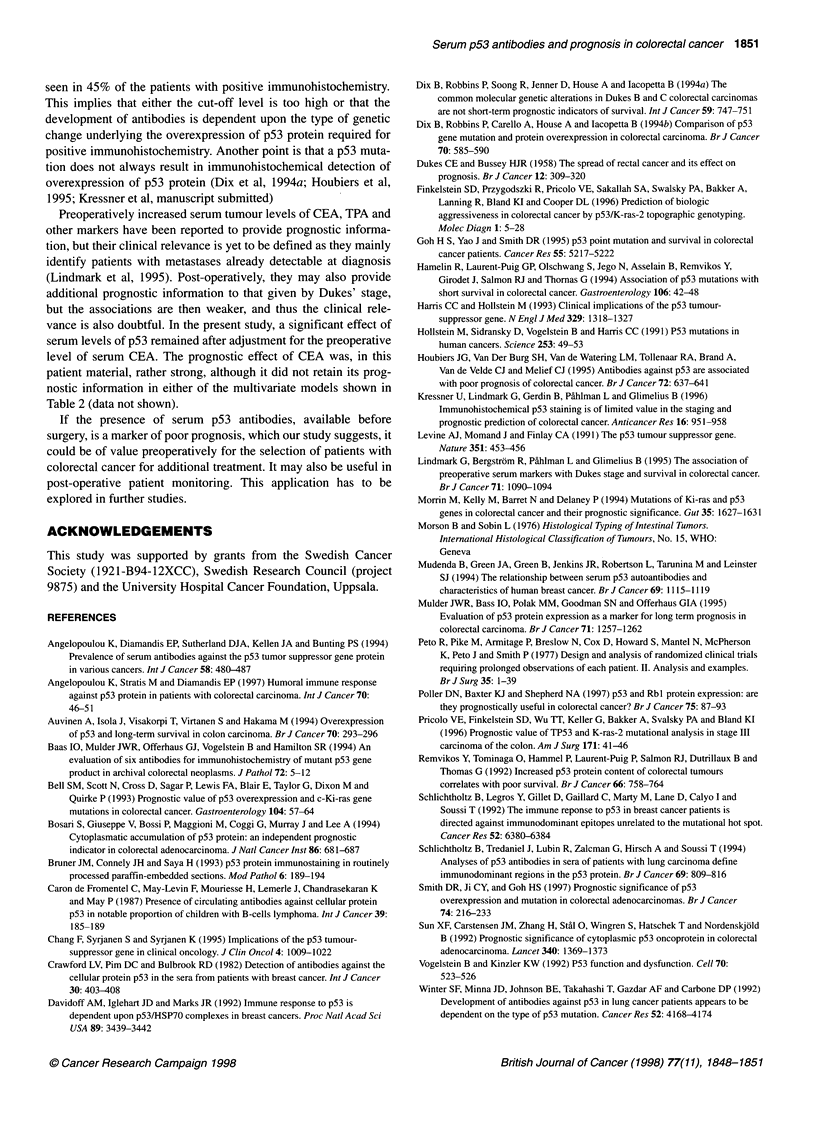# Increased serum p53 antibody levels indicate poor prognosis in patients with colorectal cancer.

**DOI:** 10.1038/bjc.1998.307

**Published:** 1998-06

**Authors:** U. Kressner, B. Glimelius, R. BergstrÃ¶m, L. PÃ¥hlman, A. Larsson, G. Lindmark

**Affiliations:** Department of Surgery, University Hospital, Uppsala, Sweden.

## Abstract

Serum p53 antibody levels were analysed using an enzyme-linked immunosorbent assay in serum samples obtained before surgery from 184 consecutive patients with primary colorectal cancer. Possible associations with tumour stage and tumour differentiation and the relation to patient survival time after a median follow-up of 6 years were studied. Analysis of serum p53 antibodies in the entire material demonstrated prognostic value in univariate analysis (P = 0.02); a finding that did not remain (P = 0.07) when the Dukes' stage was included in a multivariate analysis model. When the survival analysis was restricted to the potentially cured patients in Dukes' stages A-C, the serum p53 antibody levels retained independent prognostic value (P = 0.03). No clear association with tumour differentiation was found. We conclude that analysis of serum p53 antibodies may be of value for the identification of patients with different prognoses. This may be of relevance for selection of patients for adjuvant treatment.


British Journal of Cancer (1998) 77(11), 1848–1851
© 1998 Cancer Research Campaign

### Increased serum p53 antibody levels indicate poor prognosis in patients with colorectal cancer

**U Kressner[1], B Glimelius[2], R Bergström[3], L Påhlman[1], A Larsson[4] and G Lindmark[5]**

Departments of [1]Surgery and [2]Oncology, University Hospital, Uppsala; [3]Department of Statistics, University of Uppsala, Uppsala; [4]Department of Clinical Chemistry, University Hospital, Uppsala; [5]Department of Surgery, Umeå University Hospital, Umeå, Sweden

**Summary** Serum p53 antibody levels were analysed using an enzyme-linked immunosorbent assay in serum samples obtained before surgery from 184 consecutive patients with primary colorectal cancer. Possible associations with tumour stage and tumour differentiation and the relation to patient survival time after a median follow-up of 6 years were studied. Analysis of serum p53 antibodies in the entire material demonstrated prognostic value in univariate analysis ($P = 0.02$); a finding that did not remain ($P = 0.07$) when the Dukes' stage was included in a multivariate analysis model. When the survival analysis was restricted to the potentially cured patients in Dukes' stages A–C, the serum p53 antibody levels retained independent prognostic value ($P = 0.03$). No clear association with tumour differentiation was found. We conclude that analysis of serum p53 antibodies may be of value for the identification of patients with different prognoses. This may be of relevance for selection of patients for adjuvant treatment.

**Keywords:** serum p53 antibody; colorectal cancer; tumour stage; tumour differentiation; survival; immunohistochemistry

Mutations or other changes in the *p53* gene – the most frequent genetic alterations found in human malignancies (Levine et al, 1991; Harris and Hollstein, 1993) – can be detected in 40–70% of all colorectal adenocarcinomas (Hollstein et al, 1991; Vogelstein and Kinzler, 1992; Hamelin et al, 1994; Chang et al, 1995). It has been reported that p53 mutations are associated with poor prognosis in colorectal cancer (Hamelin et al, 1994; Goh et al, 1995; Finkelstein et al, 1996; Smith et al, 1997), while such a relation was not observed by Dix et al (1994*a*). Overexpression of the p53 protein is detectable in 30–70% of the tumours using immunohistochemistry. In some studies (Remvikos et al, 1992; Sun et al, 1992; Auvinen et al, 1994; Bosari et al, 1994) p53 protein overexpression has been shown to correlate to patient survival, while this has not been confirmed in other studies (Bell et al, 1993; Baas et al, 1994; Dix et al, 1994*a*; Morrin et al, 1994; Mulder et al, 1995; Kressner et al, 1996, Pricolo et al, 1996; Poller et al, 1997).

Mutant p53 protein, and other tumour-specific antigens, may be a target of the host's immune response (Schlichtholtz et al, 1992; Mudenda et al, 1994). Studies have shown that 9–26% of patients with different carcinomas have mounted a humoral immune response (antibodies) to abnormal p53 protein (Caron de Fromental et al, 1987; Levine et al, 1991; Angelopoulou et al, 1994). Thus, anti-p53 antibodies may be a serological marker for malignancy. Recent studies have shown increased serum antibody levels against mutant p53 protein in patients with breast (Crawford et al, 1982; Davidoff et al, 1992; Schlichtholtz et al, 1992; Mudenda et al, 1994), lung (Winter et al, 1992; Schlichtholtz et al, 1994) and colorectal cancer (Houbiers et al, 1995; Angelopoulou et al, 1997). Two of the studies in breast cancer patients have indicated that the occurrence of p53 antibodies may be a useful determinant with regard to poor prognosis (Schlichtholtz et al, 1992; Mudenda et al, 1994). This was also seen in the study on patients with colorectal cancer, but the prognostic information was limited to tumours in Dukes' stage A and Astler-Coller B1 (Houbiers et al, 1995).

The aim of this study was to evaluate whether the levels of p53 antibodies in serum samples, obtained before surgery from patients with colorectal cancer, are of importance for prediction of tumour stage and, thus, of potential value for selecting patients resected for cure to adjuvant treatment.

### MATERIALS AND METHODS

### Patients

Serum samples were collected before surgery from 184 patients resected for colorectal cancer in Uppsala and Falun County during the years from January 1987 to November 1992. The age and sex distribution, Dukes' stage and tumour differentiation are given in Table 1. One hundred and fifty-six patients (85%) were resected for cure. In the remaining 28 patients, distant metastases were detected peroperatively, and consequently they underwent a palliative resection. This group was classified as Dukes' stage D. At follow-up in October 1995, 82 (45%) patients had died from cancer or from other causes, but with known tumour burden. Twenty-eight (15%) patients died from other causes without any indications of tumour relapse. No patient was lost at follow-up. The median survival time of the 74 living patients was 80 months (range 33–102). Routine biopsies from each tumour were taken for histopathological classification. The tumours were graded according to the WHO classification (Morson and Sobin, 1976) and staged according to the Dukes' classification system (Dukes and Bussey, 1958).

*Received 18 June 1997*
*Revised 11 November 1997*
*Accepted 19 November 1997*

*Correspondence to:* U Kressner, Department of Surgery, Buskerud Central Hospital, N-3004 Drammen, Norway

**Table 1** Comparison of patients, age, gender, tumour stage and tumour differentiation in relation to increased serum p53 antibody levels in colorectal cancer

| | Cases (*n*) | p53 antibody level (*n*) | Number of cancer-related deaths | | *P*-value |
|---|---|---|---|---|---|
| | | | p53 antibody level | | |
| | | | Yes *n* (%) | No *n* (%) | |
| Age (all patients) | 184 | 59 (32) | 34 (58) | 48 (38) | |
| <70 (30–69) | 87 | 26 (30) | 16 (62) | 22 (36) | |
| ≥70 (70–89) | 97 | 33 (34) | 18 (55) | 26 (41) | NS |
| Gender | | | | | |
| Female | 89 | 26 (29) | 12 (46) | 19 (30) | |
| Male | 95 | 33 (35) | 22 (67) | 29 (47) | NS |
| Dukes' stage | | | | | |
| A | 31 | 10 (32) | 3 (30) | 3 (14) | |
| B | 84 | 26 (31) | 12 (46) | 15 (26) | |
| C | 41 | 14 (34) | 10 (71) | 13 (48) | |
| D | 28 | 9 (32) | 9 (100) | 17 (89) | NS |
| Tumour differentiation | | | | | |
| Good | 31 | 9 (29) | 6 (67) | 5 (23) | |
| Moderate | 127 | 39 (30) | 21 (54) | 35 (40) | |
| Poor | 26 | 11 (42) | 7 (64) | 8 (53) | NS |

### Serum samples and ELISA assay for anti-p53 antibodies

The serum samples were stored at –70°C until analysed. The measurements of anti-p53 were performed using enzyme-linked immunosorbent assay (ELISA) (Dianova, Hamburg, Germany). Recombinant p53 protein was coated to a microtitre plate. Peroxidase-conjugated goat anti-human IgG antibody was added to patients' sera as secondary antibody. After incubation, the optical density was determined using spectrophotometry at 450 nm. Positive control sera, containing a constant amount of anti-p53 antibodies, was obtained from Dianova. All samples were assayed in duplicate and considered positive at an optical density above the low positive control sample. We chose a p53 index of 0.01 as cut-off level as this is recommended by the manufacturer as the 'low control'.

### Immunohistochemical analysis of overexpression of p53 protein p53 protein overexpression was evaluated using an immunohistochemical method (Kressner et al, 1996). Briefly, DO-7 monoclonal antibody was used in combination with biotinylated horse anti-mouse IgG antibody as secondary antibody, followed by avidin–biotin blocking kit and ethyl carbazole substrate. The sections were classified as negative or positive.

### Statistical analyses

The Cox proportional hazards model was used in both the univariate and the multivariate survival analyses. Survival curves were constructed using the Kaplan–Meier method, and differences between curves were tested using the log-rank test (Peto et al, 1977). The chi-square test was used to test for differences in distribution between groups. *P*-values of less than 0.05 were considered statistically significant.

### RESULTS

### p53 antibodies and tumour stage, tumour differentiation and p53 immunohistochemistry

Overall, 32% (59 out of 184) of the patients had increased levels of p53 antibodies detected in their serum (Table 1). The range of the p53 antibody levels was 0.01–2.74. The proportion of p53 positivity in serum was independent of age, sex, Dukes' stage and tumour differentiation. There were similar proportions of either Dukes' stages (A–D) in both the serum-positive and the serum-negative groups. The levels of serum p53 antibodies increased with more advanced Dukes' stage, but the increase was not statistically significant (data not shown).

For 110 of the 184 patients, we stained full-cross tumour biopsy sections immunohistochemically for expression of p53 protein (wild type/mutated) with the anti-p53 antibody DO-7 (Kressner et al, 1996). The association of tumours with p53 protein overexpression with increased serum p53 antibody levels was significant: 24 (45%) of 53 patients with tumours showing overexpression had high levels of serum p53 antibodies, while this was the case in only 6 (11%) of 57 patients without p53 overexpression ($P = 0.005$).

### p53 antibodies and prognosis

There was a significant relationship between serum p53 antibody status and cancer-specific survival time, with shorter survival time for those patients who had increased levels of p53 serum antibodies than for those without. This finding was observed both when the entire patient material was analysed ($P = 0.02$, log-rank test) and when the analysis was restricted to the potentially cured patients with tumours in Dukes' stages A–C ($P = 0.03$, log-rank test; Figure 1).

An analysis based on all four Dukes' stages, and with p53 antibody status categorized as shown in Table 2, showed decreased survival with increased p53 antibody index in a univariate analysis. The relative hazards (RHs) were 1.55 (95% CI 0.78–3.07), 1.61 (0.85–3.04) and 1.98 (1.07–3.68). In a multivariate model

© Cancer Research Campaign 1998 

**Table 2** Univariate and multivariate analyses showing the effects of serum p53 antibody levels and Dukes' stage on survival in patients resected for colorectal cancer (Dukes' stages A–C). End point: disease-specific mortality. The multivariate model 1 contains only p53i and Dukes' stage. The multivariate model 2 in addition contains sex, age, localization, tumour differentiation and serum CEA levels

| | | Univariate | | | Multivariate 1 | | | Multivariate 2 | | |
|---|---|---|---|---|---|---|---|---|---|---|
| **Variable** | ***n* (dead)** | **RH** | **95% CI** | ***P*** | **RH** | **95% CI** | ***P*** | **RH** | **95% CI** | ***P*** |
| p53 | | | | | | | | | | |
| No | 106 (31) | 1.00 | (Ref.) | | 1.0 | (Ref.) | | 1.0 | (Ref.) | |
| Yes (overall) | 50 (25) | 1.94 | 1.15–3.30 | 0.03 | | | | | | |
| p53i | | | | | | | | | | |
| 0.01–0.06 | 16 (7) | 1.65 | 0.73–3.76 | 0.23 | 1.72 | 0.75–3.91 | 0.20 | 1.49 | 0.56–3.95 | 0.43 |
| >0.06–0.85 | 18 (9) | 1.82 | 0.86–3.82 | 0.12 | 2.03 | 0.96–4.28 | 0.06 | 2.28 | 1.04–5.01 | 0.04 |
| >0.85 | 16 (9) | 2.35 | 1.12–4.96 | 0.02 | 2.19 | 1.04–4.61 | 0.04 | 1.99 | 0.87–4.54 | 0.10 |
| Dukes' stage | | | | | | | | | | |
| A | 33 (4) | 1.00 | (Ref.) | | 1.0 | (Ref.) | | 1.0 | (Ref.) | |
| B | 82 (25) | 2.09 | 0.86–5.07 | 0.10 | 2.03 | 0.84–4.93 | 0.12 | 2.89 | 1.07–7.77 | 0.04 |
| C | 41 (21) | 4.16 | 1.70–10.2 | 0.002 | 4.01 | 1.62–9.92 | 0.0027 | 5.65 | 2.09–15.3 | 0.001 |

RH, relative hazard; CI, confidence interval; p53i, p53 index.

**Figure 1** (**A**) Life-table plots for all 184 patients (Dukes' stages A–D) and (**B**) the 156 patients operated for cure (Dukes' stages A–C). Group 1, patients with increased levels of serum p53 antibodies; group 2, patients without increased levels of serum p53 antibodies. —, p53(+); - - -, p53(–). O, complete responses (i.e. patients who have died from cancer); +, censored responses (i.e. patients who are alive or who have died from causes other than cancer)

adjusting for Dukes' stage, the significant effect of serum p53 levels disappeared, although the tendency that p53 antibody status was a risk factor remained ($P = 0.07$). The RHs were 1.77 (0.89–3.53), 1.74 (0.92–3.31) and 1.32 (0.70–2.49).

In the group of potentially cured patients, the prognostic value of p53 antibody status remained, however, even after adjustment for Dukes' stage (Table 2). The RH in the highest category was 2.2 (1.04–4.61). Further adjustments for a large number of variables had only marginal effects as regards the effect of p53. The RHs in the two highest categories remained at about 2, with *P*-values equal to 0.04 and 0.10.

### DISCUSSION

The proportion of patients with detectable serum p53 antibodies (32%) is consistent with a previous report on colorectal cancer (Houbiers et al, 1995), but represents a higher figure than that reported by Angelopoulou et al (1994) who found p53 antibodies in 16% of their patients.

We found that the p53 antibody status was not only a prognostic indicator in a univariate analysis but also an independent prognostic factor when analysed in a multivariate model regarding the subset of patients who were radically resected. The result is thus in contrast to Houbiers et al (1995), who did not find an independent relation with prognosis in a multivariate analysis including the Dukes' stage but reported a weak association ($P = 0.04$) between high p53 serum levels and prognosis in patients with an early stage of the disease (Dukes' stage A).

Thus, detectable levels of p53 antibodies in serum seem to indicate more aggressive tumours in colorectal cancer. Studies in other tumour types support this notion (Crawford et al, 1982; Davidoff et al, 1992; Schlichtholtz et al, 1992; Winter et al, 1992; Mudenda et al, 1994). Loss of the *p53* gene suppressor function has previously been reported in a range of human malignancies (Hollstein et al, 1991; Levine et al, 1991; Chang et al, 1995), including colorectal cancer (Levine et al, 1991; Remvikos et al, 1992; Sun et al, 1992; Vogelstein and Kinzler, 1992; Bell et al, 1993; Auvinen et al, 1994; Bosari et al, 1994; Dix et al, 1994*a*; Hamelin et al, 1994; Goh et al, 1995). A mutation in the *p53* gene results in an accumulation of mutant p53 protein or in an overproduction of normal wild-type p53 protein (Dix et al, 1994*a* and *b*). Mutant p53 protein is more stable than the wild-type p53 protein (Bruner et al, 1993; Dix et al, 1994*a* and *b*), and this seems to be necessary for the development of a humoral response with detectable levels of anti-p53 antibodies in the peripheral circulation (Crawford et al, 1982).

In this study, and in concordance with Houbiers et al (1995), we were also able to demonstrate a significant association between overexpression of p53 in tumour sections with expression of serum p53 antibodies ($P < 0.005$), although increased levels were only

 © Cancer Research Campaign 1998

seen in 45% of the patients with positive immunohistochemistry. This implies that either the cut-off level is too high or that the development of antibodies is dependent upon the type of genetic change underlying the overexpression of p53 protein required for positive immunohistochemistry. Another point is that a p53 mutation does not always result in immunohistochemical detection of overexpression of p53 protein (Dix et al, 1994*a*; Houbiers et al, 1995; Kressner et al, manuscript submitted)

Preoperatively increased serum tumour levels of CEA, TPA and other markers have been reported to provide prognostic information, but their clinical relevance is yet to be defined as they mainly identify patients with metastases already detectable at diagnosis (Lindmark et al, 1995). Post-operatively, they may also provide additional prognostic information to that given by Dukes' stage, but the associations are then weaker, and thus the clinical relevance is also doubtful. In the present study, a significant effect of serum levels of p53 remained after adjustment for the preoperative level of serum CEA. The prognostic effect of CEA was, in this patient material, rather strong, although it did not retain its prognostic information in either of the multivariate models shown in Table 2 (data not shown).

If the presence of serum p53 antibodies, available before surgery, is a marker of poor prognosis, which our study suggests, it could be of value preoperatively for the selection of patients with colorectal cancer for additional treatment. It may also be useful in post-operative patient monitoring. This application has to be explored in further studies.

### ACKNOWLEDGEMENTS

This study was supported by grants from the Swedish Cancer Society (1921-B94-12XCC), Swedish Research Council (project 9875) and the University Hospital Cancer Foundation, Uppsala.

### REFERENCES

Angelopoulou K, Diamandis EP, Sutherland DJA, Kellen JA and Bunting PS (1994) Prevalence of serum antibodies against the p53 tumor suppressor gene protein in various cancers. *Int J Cancer* **58**: 480–487

Angelopoulou K, Stratis M and Diamandis EP (1997) Humoral immune response against p53 protein in patients with colorectal carcinoma. *Int J Cancer* **70**: 46–51

Auvinen A, Isola J, Visakorpi T, Virtanen S and Hakama M (1994) Overexpression of p53 and long-term survival in colon carcinoma. *Br J Cancer* **70**: 293–296

Baas IO, Mulder JWR, Offerhaus GJ, Vogelstein B and Hamilton SR (1994) An evaluation of six antibodies for immunohistochemistry of mutant p53 gene product in archival colorectal neoplasms. *J Pathol* **72**: 5–12

Bell SM, Scott N, Cross D, Sagar P, Lewis FA, Blair E, Taylor G, Dixon M and Quirke P (1993) Prognostic value of p53 overexpression and c-Ki-ras gene mutations in colorectal cancer. *Gastroenterology* **104**: 57–64

Bosari S, Giuseppe V, Bossi P, Maggioni M, Coggi G, Murray J and Lee A (1994) Cytoplasmatic accumulation of p53 protein: an independent prognostic indicator in colorectal adenocarcinoma. *J Natl Cancer Inst* **86**: 681–687

Bruner JM, Connely JH and Saya H (1993) p53 protein immunostaining in routinely processed paraffin-embedded sections. *Mod Pathol* **6**: 189–194

Caron de Fromentel C, May-Levin F, Mouriesse H, Lemerle J, Chandrasekaran K and May P (1987) Presence of circulating antibodies against cellular protein p53 in notable proportion of children with B-cells lymphoma. *Int J Cancer* **39**: 185–189

Chang F, Syrjanen S and Syrjanen K (1995) Implications of the p53 tumour-suppressor gene in clinical oncology. *J Clin Oncol* **4**: 1009–1022

Crawford LV, Pim DC and Bulbrook RD (1982) Detection of antibodies against the cellular protein p53 in the sera from patients with breast cancer. *Int J Cancer* **30**: 403–408

Davidoff AM, Iglehart JD and Marks JR (1992) Immune response to p53 is dependent upon p53/HSP70 complexes in breast cancers. *Proc Natl Acad Sci USA* **89**: 3439–3442

Dix B, Robbins P, Soong R, Jenner D, House A and Iacopetta B (1994*a*) The common molecular genetic alterations in Dukes B and C colorectal carcinomas are not short-term prognostic indicators of survival. *Int J Cancer* **59**: 747–751

Dix B, Robbins P, Carello A, House A and Iacopetta B (1994*b*) Comparison of p53 gene mutation and protein overexpression in colorectal carcinoma. *Br J Cancer* **70**: 585–590

Dukes CE and Bussey HJR (1958) The spread of rectal cancer and its effect on prognosis. *Br J Cancer* **12**: 309–320

Finkelstein SD, Przygodszki R, Pricolo VE, Sakallah SA, Swalsky PA, Bakker A, Lanning R, Bland KI and Cooper DL (1996) Prediction of biologic aggressiveness in colorectal cancer by p53/K-ras-2 topographic genotyping. *Molec Diagn* **1**: 5–28

Goh H S, Yao J and Smith DR (1995) p53 point mutation and survival in colorectal cancer patients. *Cancer Res* **55**: 5217–5222

Hamelin R, Laurent-Puig GP, Olschwang S, Jego N, Asselain B, Remvikos Y, Girodet J, Salmon RJ and Thomas G (1994) Association of p53 mutations with short survival in colorectal cancer. *Gastroenterology* **106**: 42–48

Harris CC and Hollstein M (1993) Clinical implications of the p53 tumour-suppressor gene. *N Engl J Med* **329**: 1318–1327

Hollstein M, Sidransky D, Vogelstein B and Harris CC (1991) P53 mutations in human cancers. *Science* **253**: 49–53

Houbiers JG, Van Der Burg SH, Van de Watering LM, Tollenaar RA, Brand A, Van de Velde CJ and Melief CJ (1995) Antibodies against p53 are associated with poor prognosis of colorectal cancer. *Br J Cancer* **72**: 637–641

Kressner U, Lindmark G, Gerdin B, Påhlman L and Glimelius B (1996) Immunohistochemical p53 staining is of limited value in the staging and prognostic prediction of colorectal cancer. *Anticancer Res* **16**: 951–958

Levine AJ, Momand J and Finlay CA (1991) The p53 tumour suppressor gene. *Nature* **351**: 453–456

Lindmark G, Bergström R, Påhlman L and Glimelius B (1995) The association of preoperative serum markers with Dukes stage and survival in colorectal cancer. *Br J Cancer* **71**: 1090–1094

Morrin M, Kelly M, Barret N and Delaney P (1994) Mutations of Ki-ras and p53 genes in colorectal cancer and their prognostic significance. *Gut* **35**: 1627–1631

Morson B and Sobin L (1976) *Histological Typing of Intestinal Tumors. International Histological Classification of Tumours*, No. 15, WHO: Geneva

Mudenda B, Green JA, Green B, Jenkins JR, Robertson L, Tarunina M and Leinster SJ (1994) The relationship between serum p53 autoantibodies and characteristics of human breast cancer. *Br J Cancer* **69**: 1115–1119

Mulder JWR, Bass IO, Polak MM, Goodman SN and Offerhaus GIA (1995) Evaluation of p53 protein expression as a marker for long term prognosis in colorectal carcinoma. *Br J Cancer* **71**: 1257–1262

Peto R, Pike M, Armitage P, Breslow N, Cox D, Howard S, Mantel N, McPherson K, Peto J and Smith P (1977) Design and analysis of randomized clinical trials requiring prolonged observations of each patient. II. Analysis and examples. *Br J Surg* **35**: 1–39

Poller DN, Baxter KJ and Shepherd NA (1997) p53 and Rb1 protein expression: are they prognostically useful in colorectal cancer? *Br J Cancer* **75**: 87–93

Pricolo VE, Finkelstein SD, Wu TT, Keller G, Bakker A, Svalsky PA and Bland KI (1996) Prognostic value of TP53 and K-ras-2 mutational analysis in stage III carcinoma of the colon. *Am J Surg* **171**: 41–46

Remvikos Y, Tominaga O, Hammel P, Laurent-Puig P, Salmon RJ, Dutrillaux B and Thomas G (1992) Increased p53 protein content of colorectal tumours correlates with poor survival. *Br J Cancer* **66**: 758–764

Schlichtholtz B, Legros Y, Gillet D, Gaillard C, Marty M, Lane D, Calyo I and Soussi T (1992) The immune reponse to p53 in breast cancer patients is directed against immunodominant epitopes unrelated to the mutational hot spot. *Cancer Res* **52**: 6380–6384

Schlichtholtz B, Tredaniel J, Lubin R, Zalcman G, Hirsch A and Soussi T (1994) Analyses of p53 antibodies in sera of patients with lung carcinoma define immunodominant regions in the p53 protein. *Br J Cancer* **69**: 809–816

Smith DR, Ji CY, and Goh HS (1997) Prognostic significance of p53 overexpression and mutation in colorectal adenocarcinomas. *Br J Cancer* **74**: 216–233

Sun XF, Carstensen JM, Zhang H, Stål O, Wingren S, Hatschek T and Nordenskjöld B (1992) Prognostic significance of cytoplasmic p53 oncoprotein in colorectal adenocarcinoma. *Lancet* **340**: 1369–1373

Vogelstein B and Kinzler KW (1992) P53 function and dysfunction. *Cell* **70**: 523–526

Winter SF, Minna JD, Johnson BE, Takahashi T, Gazdar AF and Carbone DP (1992) Development of antibodies against p53 in lung cancer patients appears to be dependent on the type of p53 mutation. *Cancer Res* **52**: 4168–4174

© Cancer Research Campaign 1998